# Harnessing marine algal polysaccharides for combination cancer therapy: pharmacological mechanisms and clinical perspectives

**DOI:** 10.3389/fphar.2025.1682025

**Published:** 2025-11-25

**Authors:** Jing Zhong, Sookja Kim Chung, Xiaoling Han, Riming Huang, Baojun Xu, Io Nam Wong

**Affiliations:** 1 Faculty of Medicine, Macau University of Science and Technology, Macau, China; 2 Dr. Neher’s Biophysics Laboratory for Innovative Drug Discovery, State Key Laboratory of Mechanism and Quality of Chinese Medicine, Macau University of Science and Technology, Macau, China; 3 Guangdong Provincial Key Laboratory of Food Quality and Safety, College of Food Science, South China Agricultural University, Guangzhou, China; 4 Food Science and Technology Program, Department of Life Sciences, Beijing Normal-Hong Kong Baptist University, Zhuhai, China; 5 Institute for AI in Medicine, Faculty of Medicine, Macau University of Science and Technology, Macau, China; 6 Zhuhai MUST Science and Technology Research Institute/Macau University of Science and Technology Innovation Technology Research Institute, Hengqin, China

**Keywords:** algal polysaccharides, combination cancer therapy, anti-cancer, pharmacological effects, therapeutic safety enhancement

## Abstract

Algal polysaccharides (APs) have attracted increasing interest in recent years as adjunctive agents in cancer therapy due to their abundance, low toxicity, and diverse bioactivity. This review explores the pharmacological effects and mechanisms of APs in combination with chemotherapy, radiotherapy, and immunotherapy, as well as their potential as a nano medicine delivery system. It highlights a wide range of biological activities exhibited by APs in combination therapy, including enhanced tumor cell killing, modulation of the tumor immune microenvironment, and protection of healthy tissues from treatment-related toxicity. Furthermore, the review summarizes the advancements in the pharmacodynamics, pharmacokinetics, and safety profiles of APs, emphasizing the need for standardized raw materials, combined strengths analysis, and clinical validation as key future directions.

## 1 Introduction

Over the past few decades, despite considerable advances in cancer treatment, including chemotherapy, immunotherapy, and targeted therapy, clinical outcomes for many cancer types remain unsatisfactory. The emergence of drug resistance is a major factor contributing to therapeutic failure (Soragni et al., 2025). During treatment, cancer cells can adapt to the host environment through genetic mutations, epigenetic modifications, and alternative activation pathways, which diminish the effectiveness of standard therapies over time (He et al., 2024; Jiang et al., 2025; Tufail et al., 2024). Additionally, systemic toxicity poses a substantial limitation, especially with conventional chemotherapeutic agents. While these agents target and destroy tumor cells, they also damage normal cells, including hematopoietic stem cells, gastrointestinal mucosal cells, and neuronal cells, leading to adverse effects such as myelosuppression, gastrointestinal toxicity, and neurotoxicity (Zeien et al., 2022). Studies have shown that between 60% and 70% of cancer patients having chemotherapy and immunotherapy experience side effects (Zhang et al., 2024). This means many patients must reduce their dosage or discontinue treatment altogether. In addition, tumor cells can evade immune surveillance and destruction through various immune escape mechanisms. These challenges have prompted researchers to develop novel therapeutic strategies aimed at improving anticancer efficacy, reducing systemic toxicity, and enhancing the body’s immune response.

With the growing exploration of natural products in cancer therapy research, marine resources have emerged as a valuable reservoir of novel drug candidates due to their unique growth environments and biological activities. APs are natural bioactive polysaccharides derived from marine algae, are characterized by their abundant availability, diverse structures, and broad biological activities (Ju et al., 2023). Numerous studies have shown that APs exert antitumor effects through diverse pathways, including apoptosis induction, cell cycle arrest, preventing metastasis, and angiogenesis suppression (Liu T. et al., 2022). Additionally, APs can exert indirect cytotoxic effects on cancer by regulating the tumor immune microenvironment, including activating immune cells, modulating cytokine secretion, and enhancing immune organ function (Yao et al., 2022). As food-derived substance, APs exhibit superior biocompatibility and low toxicity compared to conventional chemotherapeutic drugs (Premarathna et al., 2025). These characteristics suggest that APs are promising antitumor candidates, and their combination with existing therapies may help overcome limitations such as severe side effects, drug resistance, and immune evasion.

**FIGURE 1:**
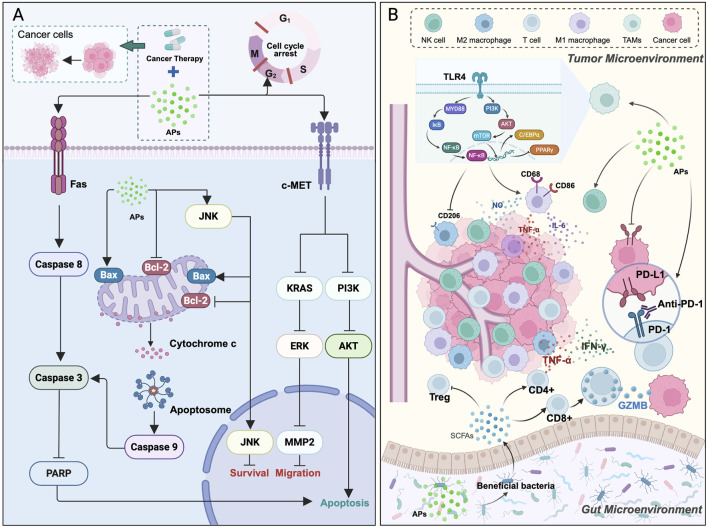
The role of APs in cancer therapy. **(A)** Direct antitumor effects of APs on tumor cells, including the inhibition of cell survival and migration and the induction of apoptosis through multiple signaling pathways (e.g., apoptosis signaling pathway, MET/KRAS/ERK, PI3K/AKT). **(B)** Indirect antitumor effects of APs mediated by modulation of the tumor microenvironment and the gut microenvironment, thereby influencing immune responses, inflammatory signaling, and other tumor-promoting processes. This schematic is original and was created based on the studies cited in the main text.

This paper discusses recent advances in APs that are being applied alongside different cancer treatments and summarizes the antitumor mechanisms of APs in combination therapy (Figure 1). The detailed literature search strategy, including inclusion and exclusion criteria, is provided in the Supplementary Material. It reviews recent findings on how these natural products may enhance the effects of chemotherapy, radiotherapy, and immunotherapy, and explores their potential role as carriers in nanodrug delivery systems (Table 1). The aim is to provide researchers and clinicians with an overview of the mechanisms, current research landscape, and potential value of APs in combined cancer treatment. Ultimately, this work hopes to facilitate the translation of these natural products from laboratory research to clinical use, providing new perspectives and directions for precision cancer therapy.

**TABLE 1:** Representative studies of algal polysaccharides in combination cancer therapies.

Source	Fraction	Cancer type	Experimental model	Effective dose	Exposure time	Cancer therapy	APs-mediated effects	Key outcomes resulted from APs	References
*Arthrospira sp.* (Family: Microcoleaceae)	Crude polysaccharides	Cholangiocarcinoma	Lymphocytes and tumor cell (KKU055, KKU213A) co-culture system	0.5, 1, 2 mg/mL	24 h	Gemcitabine	↑Cytotoxic effects by ↑ FAS and TRAIL receptor ↑The immune cell cytotoxicity against cancer cells	Crude polysaccharides promoted gemcitabine-mediated tumor cell killing while mitigating gemcitabine-induced immunosuppression	Panya et al. (2024)
*Laminaria japonica* Areschoug	Fucoidan	Hepatocellular carcinoma	Huh-7 cell Diethyl nitrosamine HCC rat model	0.15∼5 mg/mL	72 h	Sorafenib & Bevacizumab	↓The IC50 (sorafenib: 4.9 µM→0.4439 µM; bevacizumab:25.22 µM→11.55 µM) ↓Cell migration ↑The proportion of cells in S and G2/M phase ↑Cas-3, -8 ↓The level of VEGF, Ki67/CD34	Fucoidan enhanced the efficacy of sorafenib/bevacizumab by inducing apoptosis and inhibiting angiogenesis via PI3K/AKT and MAPK suppression	Abdollah et al. (2023)
*Laminaria japonica* Areschoug	FPS1M	Colorectal cancer	HCT116 xenograft model in BALB/c mice	100, 200 and 400 μg/mL	36 h	Capecitabine	↓ Ki67 ↓ CD206, ↑CD68 ↑ Bax, Cas-3, - 9, and cleaved-PARP-1/PARP-1 ↓Bcl-2 Mitigated the abnormal elevation of AST	Fucoidan enhances capecitabine sensitivity by promoting M1 macrophage polarization and mitigating liver injury	Deng et al. (2022)
*Laminaria japonica* Areschoug	OF	Breast cancer	TNBC cells 4T1 xenograft model in BALB/c mice	*in vitro*, 400 μg/mL *in vivo*, 150 mg/kg	*in vitro*,48 h *in vivo*, p.o., twice per week for 5 weeks	Olaparib	↓IL-6/p-EGFR/PD-L1 pathway Suppression of cancer stemness ↑M1 macrophage, cytotoxic T cells ↓Regulatory T cells and M2 macrophages	Fucoidan reprogrammed stemness, metabolism, and the tumor microenvironment with olaparib, preventing recurrence, metastasis, and resistance	Chen L. M. et al. (2022)
*Laminaria japonica* Areschoug	OF	Non-small cell lung cancer	Patients with non-small cell lung cancer aged 20–80	550 mg	Two times/day for 24 weeks	Cisplatin, Taxtere, Filgrastinetc.	↑ Survival rates ↑Quality of life ↑ CD19 lymphocyte population ↓Inflammatory cytokine	Fucoidan supplementation during cancer therapy enhanced 2-year survival, improved quality of life, and boosted immune function	Liu et al. (2024)
*Laminaria japonica* Areschoug	LMWF	Hepatocellular carcinoma	HBx/Src transgenic zebrafish	300 mg/kg	p.o., 3 times/week, 4 weeks	Radiotherapy	Preventing liver cell apoptosis ↓Fibrotic marker (col1a1, ctgfa, and hpse) ↓Hepatocellular carcinoma formation Modulating oxidoreductase, lipogenesis and NHEJ pathways	LMWF mitigated radiation-induced fibrosis and reduced the risk of secondary malignancies in zebrafish	Wu et al. (2020)
*Sargassum hemiphyllum* (Turner) C.Agardh	LMF	Colorectal cancer	HCT116 and Caco-2 cell	800 μg/mL	24 h	5-FU	↓Cell viability though↓MET/KRAS/ERK and PI3K/AKT↓Cell migration though ↓cMET/MMP-2 ↑Cell apoptosis ↑The proportion of cells in the S phase	LMF improved 5-FU efficacy in colorectal cancer via distinct mechanisms across wild-type and mutant KRAS.	Huang et al. (2021)
*Ecklonia cava* Kjellman	ECF	Melanoma and colon cancer	Tumour lung metastasis model	50 mg/kg	i.n., every 3 days from day 3–33	anti-PD-L1 antibody	↑Survival days ↓ Lung metastasis in B16 and CT-26 tumor models	Fucoidan enhanced anti-PD-L1 efficacy against lung metastasis by boosting dendritic cell–mediated NK and CTL activation	Zhang W. et al. (2021)
*Ascophyllum nodosum* (Linnaeus) Le Jolis *Fucus vesiculosus* Linnaeus	Fucoidan	Melanoma	C57BL/6 B16 tumor-bearing mice	8 mg/day	Diet 24 days	PD-1 antibodies	↓Tumor vulume and weight ↑CD8+ T cells and NK cells in spleen ↑Tumor infiltrating T cells ↑CD8+/CD4+ T cells ratio and NK cells in spleen	Fucoidan enhanced PD-1 antibody efficacy by activating CD8^+^ T cells through the integration of TCR/CD3–JAK-STAT pathways	Yang et al. (2021)
*Fucus vesiculosus* Linnaeus, *Undaria pinnatifida* (Harvey) Suringar*, Macrocystis pyrifera* (Linnaeus) C.Agardh	FV, UP, MP	Prostate cancer	PC3 and PBMC co-culture system	10, 50, 100 μg/mL	72 h	Nivolumab	↑Cytotoxic effects ↑PBMC proliferation	Fucoidan enhanced nivolumab efficacy by promoting PBMC proliferation and directly suppressing cancer cell growth	Park et al. (2021)
*Fucus vesiculosus* Linnaeus	Fucoidan	Breast cancers	MCF-7 and MDA-MB-231 cells	100, 200, 400 μg/mL	48 h	Doxorubicin and cisplatin	↓ Cell proliferation ↑ LC-3B ↓p62	Fucoidan enhanced doxorubicin and cisplatin sensitivity via autophagy induction	Zhang N. et al. (2021)
A group of brown algae^a^	Fucoidan	Breast cancer	BALB/c 4T1 tumor-bearing mice	200 mg/kg	p.o., once daily for 20 days	anti-PD-1 monoclonal antibody	↑Tumor inhibition rate ↑The CD8+/CD4+ ratio in tumor and spleen ↓FoxP3+ regulatory T cells in spleen ↑IFN-γ, GZMB and TNF-α ↑Beneficial bacteria: *Bifidobacterium*, *Faecalibaculum* and *Lactobacillus* *↑*Short-chain fatty acids	Fucoidan enhances the effectiveness of PD-1 in breast cancer by regulating the gut microbiota and its metabolites	Li et al. (2024)
	LF2	Pancreatic cancer	Macrophage and tumor cell (PANC-1, MIA PaCa-2) co-culture system BALB/c PANC-1-Fuc tumor-bearing mice	*in vitro*, 100, 400 μg/mL *in vivo*, 100 mg/kg/day	*in vitro*, 36 h *in vivo*, i.p., daily, 30 days	Oxaliplatin	↓Tumor volume and weight ↓The expression of Ki67 ↑ iNOS + M1 macrophage, ↓ CD206+ M2 macrophages in tumor	Fucoidan promoted M1 macrophage polarization and pro-apoptotic infiltration, thereby enhanced oxaliplatin-induced tumor killing	Deng et al. (2024)
	Fucoidan	Oral Cancer	SCC-25 cells	312.5 μg/mL	48 h	Cisplatin	↓Cell survival ↑Apoptosis markers: Cas-3, -8, -9 and PARP	Fucoidan enhances the effects of cisplatin on SCC-25 cells by inhibiting the PI3K/AKT pathway	Yang et al. (2023)
	PMGS	Cervical cancer	Hela cells	1.25∼20 mg/mL	48 h	Paclitaxel	↓Cell survival ↓Colony formation ↑ Apoptosis markers: Cleaved Cas-3, Cas-9 Inhibition of migration	PMGS enhances paclitaxel-induced apoptosis and synergistically inhibits cervical cancer cell growth and migration	Xia et al. (2023)

Across the referenced studies, untreated groups were used as negative controls, while drug-only groups served as positive controls for comparison.

^a^ These fucoidans listed are derived from brown algae (class Phaeophyceae), but the specific species were not specified in the original publications.

Abbreviations: FAS, fas cell surface death receptor; TRAIL, TNF-related apoptosis-inducing ligand; HCC, hepatocellular carcinoma; VEGF, vascular endothelial growth factor; PI3K, Phosphatidylinositol 3-Kinase; AKT, Protein kinase B; MAPK, Mitogen-activated protein kinase; FPS1M, Fucoidan polysaccharide fraction 1M; AST, alanine aminotransferase; OF, Oligo-Fucoidan; EGFR, epidermal growth factor receptor; LMF, A low-molecular-weight fucoidan; MET, Mesenchymal–epithelial transition factor; KRAS, kirsten rat sarcoma viral oncogene; ERK, extracellular signal regulated kinases; MMP2, Matrix metalloproteinase-2; ECF, *Ecklonia cava*-extracted fucoidan; NK, natural killer cell; CTL, Cytotoxic T lymphocyte; TCR, T cell receptor; JAK, janus kinase; STAT, signal transducer and activator of transcription; FV, fucoidan extracts from *Fucus vesiculosus*; UP, ucoidan extracts from *Undaria pinnatifida*; MP, fucoidan extracts from *Macrocystis pyrifera*; PC3, Prostate cancer cell line 3; PBMC, peripheral blood mononuclear cell; NHEJ, Non-homologous end joining; GZMB, Granzyme B; LF2, a sulfated fucoidan fraction from *Laminaria japonica*; PANC-1: Pancreatic carcinoma cell line 1; MIA, PaCa-2: miami pancreatic carcinoma cell line 2; PARP, Poly ADP-ribose polymerase; LC-3B, Microtubule-associated protein 1 light chain 3 beta; PMSG, polymannuroguluronate sulfate.

## 2 Structural uniqueness and antitumor relevance

In contrast to terrestrial plant and fungal polysaccharides, APs possess abundant sulfate ester groups and an unusual monosaccharide composition, notably fucose, galactose derivatives, and glucuronic acid. These unique structural features are closely associated with their pronounced biological activities (Aquino et al., 2011; Helbert, 2017). Accumulating evidence indicates that the anticancer potential of APs is largely governed by their structural characteristics, including sulfation degree, monosaccharide profile, molecular weight, and branching modifications. Highly sulfated polysaccharides show stronger anti-angiogenic and anti-tumor effects, as their negative sulfate groups interact with positively charged cell-surface proteins (e.g., EGFR, MMPs, VEGF/VEGFR), blocking tumor metastasis, angiogenesis, and invasion (Mazepa et al., 2022; Zheng et al., 2022). Low-molecular-weight APs are more easily bound by receptors on intestinal and immune cells, boosting antitumor immunity (Besednova et al., 2020; Tang et al., 2023). Their smaller size also allows them cross cell membranes more effectively, triggering apoptotic pathways (Caspase-3/8/9, PI3K/AKT/mTOR, Bax) and inducing cancer cell death (Pham et al., 2021; Song et al., 2024). Conversely, the large molecular size and high negative charge density of high-molecular-weight APs enable them to interact extensively with cationic residues and proteins on tumor cell surfaces, thereby forming a physical barrier that hinders invasion and metastasis (Hsiao et al., 2021; Luo et al., 2023; Tran et al., 2023). Furthermore, the specific monosaccharide composition of APs, including fucose, galactose, glucuronic acid, and mannose, dictates their charge distribution and sulfation patterns. These structural features modulate receptor interactions and immune activation, thereby accounting for differences in antitumor efficacy (Chen R. et al., 2022; Mazepa et al., 2022).

## 3 APs in combination cancer therapies

### 3.1 Combination with chemotherapy

Chemotherapy remains a cornerstone of cancer treatment and is one of the primary therapeutic options for various malignancies. Recent studies have shown that APs can enhance the efficacy of chemotherapeutic agents through diverse biological pathways. For example, the sulfated polysaccharide PMGS, extracted from brown algae, has shown potential as a novel sensitizer in cervical cancer. It appears to enhance the efficacy of paclitaxel by increasing cleaved caspase-3 and -9, thereby promoting caspase-dependent apoptosis (Xia et al., 2023). Additionally, Fucoidan boosted cisplatin-induced apoptosis by inhibiting the phosphatidylinositol 3-kinase (PI3K)/protein kinase B (AKT) pathway, which involves upregulating apoptotic markers such as caspase-3, -8, -9, and PARP cleavage, leading to a stronger combined inhibitory effect on oral cancer cell survival (Yang et al., 2023). Low molecular weight fucoidan (*Laminaria japonica* Areschoug) also stimulated the inhibitory effect of the fluoropyrimidine chemotherapeutic agent 5-FU on colorectal cancer cells through mechanisms like inducing S-phase cell cycle arrest and c-Jun N-terminal kinase (JNK)-mediated late apoptosis in HCT116 cells, suppressing cell survival in Caco-2 cells by modulating the c-mesenchymal–epithelial transition factor (MET)/Kirsten rat sarcoma viral oncogene homolog (KRAS)/extracellular signal-regulated kinase (ERK) and c-MET/PI3K/AKT signaling pathways, as well as inhibiting tumor cell migration in both cell types via the c-MET/matrix metalloproteinase-2 (MMP-2) pathways (Huang et al., 2021).

Moreover, APs demonstrate robust immunomodulatory activity, enabling them to modulate the tumor microenvironment and enhance the antitumor effects of chemotherapeutic agents through pharmacological mechanisms. For instance, *Spirulina sp.* (Family: Microcoleaceae) polysaccharides stimulated the cytotoxic activity of natural killer (NK) cells against cholangiocarcinoma cells, resulting in a significant reduction in cancer cell viability from 78.96% to 20.93% when combined with gemcitabine (Panya et al., 2024). Oligo-fucoidan (*Laminaria japonica* Areschoug) remodeled the tumor microenvironment by decreasing the expression of immunosuppressive factors such as PD-L1 and IL-6, which stimulates T-cell immune surveillance and promotes the polarization of tumor-associated macrophages from the immunosuppressive M2 phenotype to the antitumor M1 phenotype. This modulation synergizes with poly ADP-ribose polymerase (PARP) inhibitor Olaparib to inhibit postoperative recurrence and distant metastasis of triple-negative breast cancer (Chen L. M. et al., 2022). The regulation of macrophage polarization by APs has shown promising pharmacological potential in enhancing the efficacy of various cancer therapies. Fucoidan, such as FPS1M (*Laminaria japonica* Areschoug) and LF2, has been shown to activate the toll-like receptor 4 (TLR4) pathway, leading to the transformation of tumor-associated macrophages from the pro-tumor M2 phenotype to the antitumor M1 phenotype, and upregulating pro-inflammatory factors (iNOS, TNF-α, IL-6, and IL-12) by PI3K/AKT/mTOR or NF-κB pathways, alleviating the immunosuppressive tumor microenvironment (Deng et al., 2024; Deng et al., 2022). Combination of FPS1M with capecitabine enhances apoptotic death of colorectal cancer tumor cells (Deng et al., 2022), while LF2 combined with oxaliplatin increases M1 macrophage infiltration and antitumor activity of pancreatic cancer (Deng et al., 2024). These findings highlight the mechanistic basis and therapeutic potential of APs in synergizing with chemotherapeutic agents by modulating apoptosis and immune cell function.

Importantly, APs also provide protective effects on normal cells, which can mitigate chemotherapy-associated adverse effects. In a study using a 5-FU-induced immunosuppressive mouse model, oral administration of fucoidan restored NK cell activity, maintained IFN-γ secretion capacity, and preserved Th1 and CD11b^+^ immune cell subsets (Miyazaki et al., 2013). In patients with non-small cell lung cancer receiving chemotherapy, oligo-fucoidan (*Laminaria japonica* Areschoug) has been linked to improvements in both quality of life and overall survival. These benefits may be partly due to its ability to support immune function, specifically by increasing the proportion of CD19-positive lymphocytes and lowering inflammatory cytokine levels. (Liu et al., 2024). A clinical study has similarly highlighted the potential of fucoidan as an adjuvant therapy in standard chemotherapy for glioblastoma. The findings indicate that fucoidan not only exhibits good safety and tolerability but also improve chemotherapy related adverse effects by enhancing natural killer cell activity, suppressing the expression of pro-inflammatory cytokines (IL-1β) and improving quality-of-life scores. These insights suggest that fucoidan may play a valuable role in improving treatment adherence and survival outcomes (Kong et al., 2024).

### 3.2 Combination with radiotherapy

The off-target toxicity and the risk of secondary malignancies are the main disadvantages of radiotherapy. APs have shown potential in mitigating radiation-induced damage to normal tissues. For example, low molecular weight fucoidan (*Laminaria japonica* Areschoug) has been found to effectively alleviate hepatocellular fibrosis caused by high-dose radiation in a zebrafish model. This protective effect is mediated by downregulating lipogenic enzymes (AGPAT4, PAP, AND FASN) and cell proliferation–associated factors (CCNE1, CDK1, and CDK2), as well as modulating the expression of genes involved in redox regulation and DNA repair pathways—particularly those associated with non-homologous end joining (NHEJ)—thereby reducing the incidence of hepatocellular carcinoma (Wu et al., 2020). Although research on the combined application of APs and radiotherapy in cancer treatment is still limited, the radioprotective effects of these polysaccharides have already been identified. Fucoidan has been shown to provide hematopoietic protection in a mouse total-body irradiation model, with notable increases in survival rates, bone-marrow cell counts, and the number of endogenous spleen colonies (Lee et al., 2008). In summary, recent studies suggest that APs can effectively alleviate radiation-induced damage to tissues and cells, highlighting their potential as natural radioprotective agents. However, this area of research is still at the early stage, and the elucidation of the underlying molecular mechanisms and validation through clinical trials are still required.

### 3.3 Combination with immunotherapy

APs enhance the antitumor efficacy of immune checkpoint inhibitors (ICIs) by promoting immune cell activity, modulating the tumor microenvironment, and stimulating cytokine secretion. For instance, fucoidan extracted from *Ecklonia cava* Kjellman enhances pulmonary antitumor immunity by activating dendritic cell–mediated NK and T cell responses, significantly amplifying the inhibitory effects of PD-L1 immune checkpoint inhibitors against lung metastases of B16 melanoma and CT-26 colon cancer (Zhang W. et al., 2021). Similarly, a combination of fucoidan with PD-1 antibody therapy in melanoma not only suppresses tumor growth but also enhances the activation and effector functions of tumor-infiltrating CD8^+^ T cells (Yang et al., 2021). This combination effect appears to be mechanistically driven by fucoidan (*Fucus vesiculosus* Linnaeus) promoting T cell activation through enhanced T cell receptor (TCR)/CD3 signaling in conjunction with the JAK–STAT pathway (Yang et al., 2021). Additionally, APs can potentiate the immune response elicited by ICIs through regulation of cytokine secretion. Oral administration of fucoidan activates the gut-immune axis, stimulating immune cells to express granzyme B and secrete IFN-γ and TNF-α, thereby improving the efficacy of PD-1 monoclonal antibody treatment in breast cancer (Li et al., 2024). Various APs, including those from *Gracilaria fisheri* Xia & Abbott (Khongthong et al., 2021), *Sargassum pallidum* (Turner) C. Agardh (Gao et al., 2021), and *Ulva lactuca* Linnaeus (Abd-Ellatef et al., 2017), have been shown to stimulate the secretion of pro-inflammatory cytokines such as TNF-α, IL-1β, and IL-6. However, the mechanisms underlying their potential combination effects with immune checkpoint inhibitors remain to be further elucidated.

Although ICIs have demonstrated remarkable efficacy in various cancer types, their immune-activating mechanisms may also trigger immune overactivation, resulting in severe adverse reactions such as gastrointestinal inflammation, hepatic dysfunction, and dermatologic toxicity (Jurlander et al., 2024). As natural immunomodulators, APs not only enhance immune responses but also play a critical role in maintaining immune system balance. For example, polysaccharides derived from *Ulva prolifera* O.F. Müller (formerly *Enteromorpha prolifera*) have been shown to alleviate oxidative stress and inflammation in immune organs by inhibiting NF-κB p65 signaling and decrease pro-inflammatory cytokines (TNF-α and IL-1β) (Liu W. C. et al., 2022). Similarly, fucoidan appears to have a dual effect. Supplementation with fucoidan can activate resting macrophages but inhibit the production of NO and proinflammatory factors in macrophages active by TLR agonists (LPS, Pam3CSK4) (Miyazaki et al., 2022). In a dextran sulfate sodium-induced mouse model of intestinal inflammation, polysaccharides from *Gracilaria lemaneiformis* (Bory) Greville mitigate inflammation by downregulating TNF-α, IL-6, and IL-1β (Han et al., 2021). Moreover, polysaccharides derived from algae such as *Gracilaria fisheri* Xia & Abbott (Charoensiddhi et al., 2022), *Ulva pertusa* Kjellman (formerly *Enteromorpha pertusa*) (Son et al., 2024), and *Phaeodactylum tricornutum* Bohlin (Ye et al., 2025) have been shown to modulate gut microbiota composition and reduce intestinal permeability, alleviating gastrointestinal adverse effects, a particularly beneficial property in combination therapies involving cytotoxic T-lymphocyte antigen 4 (CTLA-4) and PD-1 inhibitors, which are frequently associated with high gastrointestinal toxicity. However, research on the role of APs in mitigating immune-related toxicity remains limited. Most existing studies have focused on their ability to enhance the antitumor efficacy of immune checkpoint inhibitors, with fucoidan being the most extensively studied. However, the mechanisms by which other types of APs contribute to immune homeostasis and alleviate immunotherapy, related adverse effects remain largely unexplored. This underscores the urgent need for additional data to support their clinical relevance.

### 3.4 Nanocarriers/Drug delivery systems

Recently, nanotechnology-based strategies for cancer therapy have advanced rapidly, with natural polymeric materials emerging as key components in the design of drug delivery systems due to their excellent biocompatibility and modifiability. APs, particularly fucoidan, alginate, and chitosan, have received considerable attention. Their natural origin, high safety profile, biodegradability, structural diversity, and abundance of functional groups make them ideal for constructing nanodrug delivery platforms. These materials offer distinct benefits in combination cancer treatments, improving therapeutic outcomes while minimizing systemic toxicity (Wang et al., 2024). Firstly, their excellent biocompatibility and biodegradability ensure safe for *in vivo* application and effectively minimize systemic toxicity (Hao et al., 2021). Secondly, they possess high water solubility and physicochemical stability, enabling controlled drug release in response to varying pH levels and enzymatic environments (Barbosa et al., 2019; Coutinho et al., 2020). Additionally, the abundance of functional groups, including carboxyl, hydroxyl, and sulfate groups, enabling the attachment of targeting ligands or stimuli-sensitive components. This enables precise drug delivery triggered by specific cues such as pH, enzymatic activity, or temperature (Lu et al., 2017). Numerous evidence suggests that APs, functioning either as encapsulation matrices or surface-modifying agents, offer effective delivery platforms for various anticancer drugs, including cisplatin, doxorubicin, and paclitaxel. For example, fucoidan-modified nanomaterials have been engineered to actively target tumors with elevated post-radiotherapy expression of P-selectin, leveraging fucoidan’s natural affinity for P-selectin receptors on tumor cell surfaces (DuRoss et al., 2021). This strategy significantly enhances drug accumulation at the tumor site and improves the efficacy of chemotherapy. Similarly, alginate-based hydrogels facilitate the sustained release of paclitaxel, increasing therapeutic effectiveness while decreasing systemic toxicity (Nazemi et al., 2020). Moreover, nanoparticles co-loaded with fucoidan, sodium alginate, and chemotherapeutics (e.g., paclitaxel and doxorubicin) have been shown to overcome multidrug resistance and improve drug internalization efficiency (Jafari et al., 2020; Qiongyan et al., 2025). These algal polysaccharide-based delivery systems not only provide physical protection to the drug and extend its circulation time but also enhance therapeutic benefits through their inherent antitumor and immunomodulatory properties. In summary, APs represent a versatile and valuable component of drug delivery systems, offering integrated advantages in combination cancer therapy, including efficient delivery, tumor targeting, sustained release, and therapeutic enhanced therapeutic efficacy, with strong potential for future applications in precision medicine and smart nanotherapeutics.

## 4 Preclinical and clinical progress

APs have shown notable promise as natural antitumor candidates in preclinical research. Advances in extraction techniques, such as enzyme-assisted extraction, supercritical fluid extraction, and microbial fermentation, coupled with purification methods like ethanol precipitation combined with chromatography or ultrafiltration with ion-exchange, along with chemical modifications (e.g., sulfation, phosphorylation, and selenization), have significantly improved the preparation efficiency, solubility, and biological activity of APs. These emerging techniques establish a solid foundation for subsequent pharmacodynamic studies (Zhou and Li, 2024).

Although pharmacokinetic data on APs are still limited, existing studies suggest that these natural products generally exhibit rapid distribution, short half-lives, and low oral bioavailability (Chudasama et al., 2021; Pozharitskaya et al., 2018). These limitations are primarily due to their large molecular weight and high polarity, which hinder efficient absorption and systemic circulation. Nevertheless, strategies such as chemical derivatization, enzymatic hydrolysis to produce oligosaccharides, and integration into nanocarrier systems have proven effective in improving pharmacokinetic properties, facilitating enhanced accumulation in tumors or target tissues (Chakraborty et al., 2025; Venturini et al., 2025). Importantly, these optimizations not only improve the systemic bioavailability of APs but also enhance their antitumor efficacy when used in combination with chemotherapy, radiotherapy, or immunotherapy (Chakraborty et al., 2025; Zhou and Li, 2024). Benefits include prolonged circulation time, increased intratumoral drug concentration, and reduced resistance to therapy (Venkatesan et al., 2016). To facilitate clinical translation, it is crucial to perform thorough pharmacokinetic profiling and to identify APs with optimal absorption and biodistribution characteristics.

Regarding toxicological safety, studies assessing acute, subacute/subchronic, genotoxicity, and cytotoxicity have demonstrated that APs from various sources exhibit low toxicity toward healthy organs. Potential risks primarily arise from high doses, which may induce biochemical disturbances or lead to indirect effects due to interactions with co-administered drugs or metal ions. In conclusion, while further research is warranted, current preclinical studies of APs encompass a broad spectrum, from structural modification and pharmacodynamic evaluation to drug formulation, providing a robust scientific basis for their future clinical development.

## 5 Limitations

Although growing evidence supports the potential benefits of combining algal polysaccharides with conventional cancer therapies, current research still faces several limitations. First, numerous studies have used crude, unpurified APs with complex compositions and inadequate structural characterization, making it difficult to clarify structure–activity relationships. Additionally, some studies used commercial fucoidan products (as shown in Table 1 under a group of brown algae), insufficient information about the algal source (species, family, genus, and location), which limits the reproducibility and comparability of the research. However, fucoidans from different sources generally share similar sulfated fucose backbone structures and monosaccharide compositions, which largely determine their biological activities. Meanwhile, fucoidan has shown prominent effects in combination cancer therapy. Therefore, even without complete taxonomic information, these studies still offer valuable insights into the overall pharmacological potential of APs and are essential for accurately reflecting the current progress in this research field. Second, many studies have employed *in vitro* cell models or immunodeficient mouse models, both of which inadequately represent the dynamic architecture of the tumor immune microenvironment. The absence of humanized or clinically pertinent animal models capable of evaluating host–tumor interactions markedly constrain the translational applicability of current findings. Third, existing studies generally assess antitumor efficacy using cell viability or tumor inhibition rates, but rigorous combination index analyses are rarely performed. Consequently, reported combination effects remain largely qualitative rather than quantitatively validated. Fourth, mechanistic investigations largely remain at the level of signaling pathway correlations, without functional verification of molecular targets, such as gene knockout, overexpression, or inhibition assays, thereby hindering the elucidation of the fundamental molecular basis of APs-induced antitumor activity. Finally, systematic pharmacokinetic and toxicological studies remain limited, and no large-scale, rigorously controlled clinical trials have confirmed its efficacy and safety.

## 6 Challenges and future directions

Considering these existing limitations, several key challenges must be addressed to advance the development and application of APs-based combination cancer therapies. First, the diversity of sources, preparation methods, and degrees of chemical modification leads to instability in pharmacological activity and safety, thereby limiting clinical translation and industrialization. Establishing a multi-level control system covering raw materials, chemical properties, safety, preparation processes and functional evaluation is essential. Furthermore, incorporating glycomics technologies and minimum information required for a glycomics experiment (MIRAGE) guidelines could facilitate the creation of standardized databases and protocols, ensuring product consistency, quality assurance, and regulatory compliance (York et al., 2014). Second, existing clinical studies remain small in scale and lack rigorous design. Most existing trials remain small in scale, lack standardization, and are not supported by comprehensive pharmacokinetic or toxicological data. Future research should prioritize large-scale, multi-center, randomized controlled trials with harmonized formulations and quality standards. Patient stratification strategies should be incorporated, while outcome measures should extend beyond therapeutic efficacy to include improvements in quality of life and the reduction of adverse effects. Finally, although APs have been shown to enhance the efficacy of various cancer therapies, the detailed molecular mechanisms underlying these effects are still not well characterized. Future studies should employ multi-omics technologies (e.g., transcriptomics, metabolomics, single-cell sequencing) integrated with bioinformatics to systematically map target networks, signaling pathways, and inter-patient variability in treatment response. Moreover, the intrinsic modifiability and self-assembly properties of APs offer unique opportunities in drug delivery, targeted therapy, and the design of stimuli-responsive carriers. When combined with nanotechnology, biomaterials, and 3D printing, these materials may be developed into advanced controlled-release systems, enabling more precise, efficient cancer therapies. Collectively, quality standardization, rigorous clinical validation, mechanistic exploration, and integration with emerging technologies will be key to advancing APs from supportive adjuvants to integral components of precision oncology.
