# Supplementary material for: Harnessing marine algal polysaccharides for combination cancer therapy: pharmacological mechanisms and clinical perspectives
Source: Front Pharmacol. 2025 Nov 25;16:1682025. doi: 10.3389/fphar.2025.1682025 (PMC12685836; doi:10.3389/fphar.2025.1682025)
Supplement: Supplementary file 1 [file Supplementaryfile1.docx]

1. Literature Search Strategy and Study Selection Criteria

Literature searches were conducted in PubMed, Web of Science, and Google Scholar for articles published up to March 2025 using the keywords: “algal polysaccharide AND (cancer OR neoplasm* OR tumor OR carcinoma OR malignant*)”. The inclusion criteria were as follows: (i) studies related to cancer; (ii) studies focusing on polysaccharides derived from algae; (iii) studies involving combination with chemotherapy, radiotherapy, immunotherapy, or drug/nanomaterial delivery systems; and (iv) *in vitro*, animal, or clinical studies. Exclusion criteria included studies unrelated to cancer, studies not involving algal polysaccharides.
